# Supplementary material for: Nationwide and long-term epidemiological research of snakebite envenomation in Taiwan during 2002–2014 based on the use of snake antivenoms: A study utilizing National Health Insurance Database
Source: PLoS Negl Trop Dis. 2023 Jun 8;17(6):e0011376. doi: 10.1371/journal.pntd.0011376 (PMC10249856; doi:10.1371/journal.pntd.0011376)
Supplement: S1 Table — (DOCX) [file pntd.0011376.s001.docx]

Table S1. Characteristics of patients who received different antivenoms in Taiwan (*N* = 12,542)

| Variables | | FH  *n* = 9,194 | | FN  *n* = 2,150 | | FA  *n* = 81 | | FD  *n* = 11 | | Mixed  *n* = 1,106 | |
| --- | --- | --- | --- | --- | --- | --- | --- | --- | --- | --- | --- |
|  |  | *n* | % | *n* | % | *n* | % | *n* | % | *n* | % |
| Gender | Male | 6,365 | 69.2% | 1,636 | 76.1% | 68 | 84.0% | 11 | 100.0% | 711 | 64.3% |
|  | Female | 2,688 | 29.2% | 476 | 22.1% | 12 | 14.8% | 0 | 0.0% | 161 | 14.6% |
|  | Unknown | 141 | 1.5% | 38 | 1.8% | 1 | 1.2% | 0 | 0.0% | 27 | 2.4% |
| Age | <18 years | 327 | 3.6% | 71 | 3.3% | 2 | 2.5% | 1 | 9.1% | 58 | 5.2% |
|  | 18-64 years | 6,425 | 69.9% | 1,656 | 77.0% | 67 | 82.7% | 8 | 72.7% | 802 | 72.5% |
|  | ≥65 years | 2,442 | 26.6% | 423 | 19.7% | 12 | 14.8% | 2 | 18.2% | 246 | 22.2% |
| Charlson comorbidity index | 0 | 2,334 | 25.4% | 534 | 24.8% | 25 | 30.9% | 2 | 18.2% | 301 | 27.2% |
|  | 1 | 1,883 | 20.5% | 446 | 20.7% | 20 | 24.7% | 4 | 36.4% | 212 | 19.2% |
|  | 2 | 1,497 | 16.3% | 321 | 14.9% | 15 | 18.5% | 1 | 9.1% | 175 | 15.8% |
|  | ≥3 | 3,480 | 37.9% | 849 | 39.5% | 21 | 25.9% | 4 | 36.4% | 418 | 37.8% |
| Year | 2002 | 757 | 8.2% | 204 | 9.5% | 10 | 12.3% | N/A | N/A | 101 | 9.1% |
|  | 2003 | 731 | 8.0% | 181 | 8.4% | 7 | 8.6% | N/A | N/A | 100 | 9.0% |
|  | 2004 | 588 | 6.4% | 173 | 8.0% | 11 | 13.6% | N/A | N/A | 99 | 9.0% |
|  | 2005 | 633 | 6.9% | 178 | 8.3% | 8 | 9.9% | N/A | N/A | 89 | 8.0% |
|  | 2006 | 722 | 7.9% | 170 | 7.9% | 7 | 8.6% | N/A | N/A | 98 | 8.9% |
|  | 2007 | 721 | 7.8% | 197 | 9.2% | 6 | 7.4% | N/A | N/A | 86 | 7.8% |
|  | 2008 | 748 | 8.1% | 197 | 9.2% | 7 | 8.6% | 0 | 0.0% | 82 | 7.4% |
|  | 2009 | 681 | 7.4% | 154 | 7.2% | 6 | 7.4% | 3 | 27.3% | 79 | 7.1% |
|  | 2010 | 715 | 7.8% | 146 | 6.8% | 7 | 8.6% | 0 | 0.0% | 76 | 6.9% |
|  | 2011 | 692 | 7.5% | 140 | 6.5% | 3 | 3.7% | 3 | 27.3% | 73 | 6.6% |
|  | 2012 | 735 | 8.0% | 128 | 6.0% | 2 | 2.5% | 3 | 27.3% | 77 | 7.0% |
|  | 2013 | 754 | 8.2% | 149 | 6.9% | 6 | 7.4% | 1 | 9.1% | 67 | 6.1% |
|  | 2014 | 717 | 7.8% | 133 | 6.2% | 1 | 1.2% | 1 | 9.1% | 79 | 7.1% |
| Season | Spring | 2,225 | 24.2% | 460 | 21.4% | 18 | 22.2% | 0 | 0.0% | 212 | 19.2% |
|  | Summer | 3,002 | 32.7% | 987 | 45.9% | 40 | 49.4% | 7 | 63.6% | 463 | 41.9% |
|  | Fall | 2,982 | 32.4% | 599 | 27.9% | 17 | 21.0% | 3 | 27.3% | 338 | 30.6% |
|  | Winter | 985 | 10.7% | 104 | 4.8% | 6 | 7.4% | 1 | 9.1% | 93 | 8.4% |
| Region | Northern | 3,299 | 35.9% | 483 | 22.5% | 25 | 30.9% | 1 | 9.1% | 518 | 46.8% |
|  | Central | 1,235 | 13.4% | 462 | 21.5% | 10 | 12.3% | 0 | 0.0% | 156 | 14.1% |
|  | Southern | 2,130 | 23.2% | 846 | 39.3% | 13 | 16.0% | 3 | 27.3% | 226 | 20.4% |
|  | Eastern | 2,530 | 27.5% | 359 | 16.7% | 33 | 40.7% | 7 | 63.6% | 206 | 18.6% |
| Occupation | Agricultural worker | 3,856 | 41.9% | 795 | 37.0% | 24 | 29.6% | 5 | 45.5% | 365 | 33.0% |
|  | Laborer | 2,184 | 23.8% | 579 | 26.9% | 20 | 24.7% | 1 | 9.1% | 324 | 29.3% |
|  | Others | 3,154 | 34.3% | 776 | 36.1% | 37 | 45.7% | 5 | 45.5% | 417 | 37.7% |
| Anaphylaxis treated with epinephrine | | 13 | 0.1% | 2 | 0.1% | 0 | 0.0% | 0 | 0.0% | 2 | 0.2% |
| Infection treated with antibiotic | | 1,862 | 20.3% | 503 | 23.4% | 13 | 16.0% | 3 | 27.3% | 335 | 30.3% |
| Surgery | | 448 | 4.9% | 301 | 14.0% | 13 | 16.0% | 0 | 0.0% | 152 | 13.7% |
| Neurological complications | | 95 | 1.0% | 68 | 3.2% | 2 | 2.5% | 0 | 0.0% | 15 | 1.4% |
| Endotracheal intubation | | 18 | 0.2% | 46 | 2.1% | 2 | 2.5% | 0 | 0.0% | 17 | 1.5% |
| Hospitalization | | 3,479 | 37.8% | 797 | 37.1% | 30 | 37.0% | 3 | 27.3% | 593 | 53.6% |
| Inter-hospital transfer | | 2,748 | 29.9% | 713 | 33.2% | 26 | 32.1% | 5 | 45.5% | 476 | 43.0% |
| Death | | 7 | 0.1% | 6 | 0.3% | 0 | 0.0% | 0 | 0.0% | 1 | 0.1% |

Abbreviations: FH, bivalent antivenom against *T. s. stejnegeri* and *P. mucrosquamatus*; FN, bivalent antivenom against *N. atra* and *B. m. multicinctus*; FA, antivenom against *D. acutus*; FD, antivenom against *D. siamensis*; N/A, not applicable.
