# Supplementary material for: Nationwide and long-term epidemiological research of snakebite envenomation in Taiwan during 2002–2014 based on the use of snake antivenoms: A study utilizing National Health Insurance Database
Source: PLoS Negl Trop Dis. 2023 Jun 8;17(6):e0011376. doi: 10.1371/journal.pntd.0011376 (PMC10249856; doi:10.1371/journal.pntd.0011376)
Supplement: Attachment — Submitted filename: 20230505 point to point response.docx [file pntd.0011376.s003.docx]

**Point to point response**

The manuscript adds important information to the field. It will be great to add actual epidemiological data on snakebite envenomation rather than the surrogates used.
Because of its importance I recommend that the authors revise the manuscript after major revision and resubmit.
Reviewer's Responses to Questions
<b>Key Review Criteria Required for Acceptance?</b></br></br>
As you describe the new analyses required for acceptance, please consider the following:</br></br>
<b>Methods</b></br></br>
-Are the objectives of the study clearly articulated with a clear testable hypothesis stated?</br>
-Is the study design appropriate to address the stated objectives?</br>
-Is the population clearly described and appropriate for the hypothesis being tested?</br>
-Is the sample size sufficient to ensure adequate power to address the hypothesis being tested?</br>
-Were correct statistical analysis used to support conclusions?</br>
-Are there concerns about ethical or regulatory requirements being met?</br></br>
Reviewer #1: Please refer the comments under Summary and General Comments

Reviewer #2: -the incidence of venomous snakebite being extrapolated from hospital insurance payouts maybe fallacious as many of the envenomed may actually land up with alternate therapies which fall below the insurance radar as so commonly happens in South-East Asian countries and whether these numbers reporting for treatment in modern medical facilities are thus truly representative enough
- it is not stated as to whether the e Taiwan National Health Insurance covers for all individuals and all diseases or in short whether the health system is uniformly insured for all.

Response: Thank you for the valuable comments. The NHI system is mandatory for people and provides uniform insurance coverage for all; therefore, we have revised the sentences in the Study population and setting section to help readers better understand the NHI.

- There would be instances of victims being given ASV for a presumptive bite which would finally turn out as non-venomous or other bites which could only be confirmed with study of the individual case notes

Response: Thank you for the valuable comments. As per the suggestions of Reviewer 1, we provided the diagnosis procedure of venomous snakebites in accordance with the guidelines of the Taiwan Poison Control Center in the Introduction section. We agree with your comments that a very small number of individuals who received snake antivenom may have ultimately been diagnosed with non-venomous snakebites or other types of bites. Therefore, we have added additional sentences to the Limitations section. Furthermore, we provided an explanation that alternative therapies for SBE are not commonly practiced in Taiwan because traditional healers are illegal in Taiwan, where all medical practices are regulated by laws, to help readers understand the medical conditions in Taiwan in the new first paragraph of Discussion section.

<b>Results</b></br></br>
-Does the analysis presented match the analysis plan?</br>
-Are the results clearly and completely presented?</br>
-Are the figures (Tables, Images) of sufficient quality for clarity?</br></br>
Reviewer #1: Please refer the comments under Summary and General Comments

Reviewer #2: The incidence of venomous snakebite may not be truly representative but a pointer towards the results given

Response: Thank you for the comment.
--------------------
<b>Conclusions</b></br></br>
-Are the conclusions supported by the data presented?</br>
-Are the limitations of analysis clearly described?</br>
-Do the authors discuss how these data can be helpful to advance our understanding of the topic under study?</br>
-Is public health relevance addressed?</br></br>
Reviewer #1: Please refer the comments under Summary and General Comments
Reviewer #2: Reasonably well, yes
--------------------
<b>Editorial and Data Presentation Modifications?</b></br><br/>
Use this section for editorial suggestions as well as relatively minor modifications of existing data that would enhance clarity. If the only modifications needed are minor and/or editorial, you may wish to recommend “Minor Revision” or “Accept”.
Reviewer #1: None
Reviewer #2: this manuscript on a Neglected Tropical disease is presented well given that it has all the negatives that come alongside retrospective data analysis using an Insurance database
I would think it could be accepted with revisions and of modest interest to readers

Response: Thank you.
--------------------
<b>Summary and General Comments</b></br></br>
Use this section to provide overall comments, discuss strengths/weaknesses of the study, novelty, significance, general execution and scholarship. You may also include additional comments for the author, including concerns about dual publication, research ethics, or publication ethics. If requesting major revision, please articulate the new experiments that are needed.

Reviewer #1: Suggest to change the “snake envenomation” as “snakebite envenomation” in the title and throughout the manuscript.
The terms “envenoming” and envenomation” have been used is different parts of the manuscript. Suggest to use single terminology throughout the manuscript.

Response: “Snakebite envenomation” (SBE) is used in the title and throughout the manuscript.

Abstract: Reword the introduction of the abstract to highlight the lack of long-term epidemiological data of snakebite envenomation (SBE). It is not necessary to include the names of the venomous snakes with in the limited word count in the abstract.

This is not a true prospectively conducted SBE epidemiology study. All epidemiological results were generated based on the distribution and used of different antivenoms in different parts of Taiwan. Therefore, reword this sentence “This study aimed to explore the epidemiology of snake envenomation in Taiwan to facilitate the development of prevention strategies and resource allocation.” to highlight that this study is based on Antivenom data.

Response: Thank you for the comments. The introduction of abstract has been revised based on your suggestions.

Add thousand separators in all the numerical values more than 999. (throughout the manuscript).
Response: A separator is used in all values above 999.

Authors summary: Include the common names of the listed snake species.

Response: The common names are provided.

Authors summary: “The low case-fatality rate observed might be due to easy access to modern medicine, immediate availability of antivenoms in healthcare facilities, few adverse effects of antivenoms, and prompt surgical intervention.”
Lack of highly venomous snakes have highly potent venom is a leading factor for low case fatality. Please include that in this sentence.
Response: Thank you for the valuable comments. This sentence was added.

Under introduction: Following details on Taiwan and related to SBE have to be included.
Total land area, total population, proportion of people in rural areas, proportion of people in agricultural sector, type of snake antivenom used, antivenom manufacturers, details of the snakebite management guidelines used to treat patients, basis of deciding type of antivenom, how snakes are identified in hospitals.
Response: Thank you for the comments. The third paragraph of the introduction was carefully revised to address this information. The government has statistic data of agricultural population but not for population in rural areas; therefore, we provided the former in this paragraph.

At the end of introduction: Aims and objectives of this study have not been stated.
Response: The study aims and objectives were added.

Under study population and setting: include the details of antivenom manufacturers.
Response: The detail of the only manufacturer of snake antivenom in Taiwan has been added in the Introduction section based on your suggestions as follows: “ The Taiwan Centers for Disease Control (CDC), the only snake antivenom manufacturer, produces four types…”.

Under study population and setting: “We assumed that patients who received specific snake antivenom were envenomed by relevant snakes”. Majority of the data analysis of this study depends on accurate identification of snake bitten by and correct used of antivenom. Therefore, it is important to provide more details about how bitten snakes were identified and antivenom selection procedure.
Response: Thank you for the valuable comments. This information was added in the new third paragraph of Introduction section based on your previous suggestions. As a result, the sentence “We assumed…” was removed. Additionally, since no commercial venom serum test was available, the limitations of the diagnostic procedures for SBE that adhere to the Taiwan Poison Control Center flowchart were included in the Limitations section.

The presented epidemiological data on SBE is based on antivenom usage database and which is far from the actual SBE epidemiology described by a prospective observation study. There is a possibility of some envenomated patients may not seek hospital admissions, some patients may not give the antivenom, some patients may have received antivenom inappropriately. Therefore, I would like to suggest to present this data as “use of snake antivenom in Taiwan”. This factor has not been highlighted because of using “epidemiology of snake envenomation” in several places. I suggest to reword the title and other relevant sections accordingly.

Response: Thank you for the comments. We have reworded the title, abstract, author summary, introduction, and relevant sections to highlight that this study is based on the use of antivenom.

Discussion: Highlight that all these epidemiological data is based on the Antivenom distribution and used data and the actual SBE epidemiology would be somewhat different and explain the reasons for the difference.
Response: Thank you for the great comments. As per your previous suggestion, we has emphasized the study was based on the use of snake antivenom in Taiwan in relevant sections. We also revised the first paragraph of the discussion to highlight these issues.

Provide some SBE epidemiology figures in some Asian countries and discuss the Taiwan values with the values of some Asian countries.

Response: Thank you for the comments. In accordance with your previous comments, we have revised the first paragraph of Discussion.

Limitations:
Describe the issues of accurate snake identification and decision of use of different type of Antivenom based on that.
Response: Thank you for the valuable comments. We provided the diagnostic approach for snake ID in the Introduction section per your suggestions. As no commercial venom serum test was available in Taiwan, we further addressed the issue that the decision to use a different type of antivenom for snakebite treatment, based solely on the snake identification procedure, could be a limitation of our study. Therefore, the study findings should be interpreted with caution.

“Second, the epidemiology of individual snakebites….” Here this is not on snakebites, snakebite envenomation based on antivenom using data.
Response: The sentence has been reworded.

Conclusions: Second line “clinical features”
This study has not described the detailed clinical features of SBE. Presented overall local and systemic effects such as neurotoxicity, renal injury, and so on. Hence, this has to be modified as “local and overall systemic toxin effects”

Response: The sentence has been reworded.

Table 1. Caption: “Characteristic distributions of venomous snakebites, and calculation of…”
This is not snakebites, this has to be modified as “Characteristic distributions of SBE cases based on use of snake antivenom, and calculation of….
Response: The caption was reworded.

**Reviewer #2:** I read the manuscript written by Jen-Yu Hsu et al with a lot of interest and have the following remarks to make
-in the author summary he states that venomous snakebite is a tropical disease and should not be neglected, whilst venomous snakebite is already listed as a neglected tropical disease (NTD)

Response: Thank you very much. The sentence has be reworded.

- Bungarus multicinctus multicinctus is I believe not distributed in India

Response: This sentence has been deleted.

- ‘Wound infection and antibiotic use were defined as having erysipelas, cellulitis, fasciitis, or myositis and receiving oral or intravenous antibiotics within 7 days, respectively’, would not hold true for all fasciitis or myositis as a lot of it could be attributed directly to the venoms of viperid, crotalid and even elapid species as well

Response: Thank you for the valuable comments. We agree with you that diagnosing wound infections in SBEs can be difficult and may be overestimated if deep tissue or biopsy cultures are not performed for the wound. However, we observed that 76.8% to 80.9% of N. atra bite patients developed wound infections in studies conducted by Mao et al. Out of the 59 and 87 patients, 50 and 73, respectively, had positive wound bacterial cultures [1,2]. In addition, 22 out of 27 and 30 out of 33 had positive deep tissue or biopsy cultures, respectively [2,3]. Furthermore, necrotizing soft tissue infections, including necrotizing fasciitis, were observed in 42.1% to 44.7% of N. atra bites [2,3]. On the other hand, wound infections are less common in T. s. stejnegeri and P. mucrosquamatus (crotaline) bites, with rates of 11.4% and 27.4%, respectively [4,5]. In this study, which utilized the National Health Insurance database, we used criteria similar to those in a recent report to identify SBE wound infections [6]. Our findings indicate that 20.3% of patients who received FH antivenom (i.e., antivenom for T. s. stejnegeri and P. mucrosquamatus) developed wound infections, which is consistent with previous studies conducted by Mao et al. [4,5], and lower than the study conducted by João Victor Soares Coriolano Coutinho et al. [6]. Although the incidence of wound infection might still have been overestimated in this study, we believed the overestimate was likely to be of limited magnitude.

- I wonder how many of the envenomed actually land up with alternate therapies which fall below the insurance radar as so commonly happens in South-East Asian countries and whether these numbers reporting for treatment in modern medical facilities are thus truly representative enough

Response: Thank you for the valuable comments. We addressed these issues based on Reviewer 1's suggestions in the first paragraph of the discussion. In short, alternative therapies for SBE do not exist or are extremely rare in Taiwan. Traditional healers are illegal because all medical practices are regulated by laws. Therefore, we believe that our study findings are representative for individuals with SBE in Taiwan.

- Given the constraints placed by retrospective analysis of data and that it being based on insurance pay-outs I think that the manuscript is structured well and makes for a good read.
Response: Thank you very much.
--------------------

PLOS authors have the option to publish the peer review history of their article (<a href="<https://journals.plos.org/plosntds/s/editorial-and-peer-review-process#loc-peer-review-history>" target="_blank">what does this mean?</a>). If published, this will include your full peer review and any attached files.<br><br>

If you choose “no”, your identity will remain anonymous but your review may still be made public.<br><br>

<b>Do you want your identity to be public for this peer review?</b> For information about this choice, including consent withdrawal, please see our <a href="<https://www.plos.org/privacy-policy>" target="_blank">Privacy Policy</a>.

Reviewer #1: Yes: Kalana Maduwage
Reviewer #2: Yes: Jaideep Menon

**References**

1.	Mao YC, Liu PY, Hung DZ, et al. Bacteriology of Naja atra Snakebite Wound and Its Implications for Antibiotic Therapy. Am J Trop Med Hyg. 2016 May 4;94(5):1129-35.

2.	Mao Y-C, Liu P-Y, Chiang L-C, et al. Naja atra snakebite in Taiwan. Clinical Toxicology. 2018;56(4):273-280.

3.	Lai C-S, Liu P-Y, Lee C-H, et al. The development of surgical risk score and evaluation of necrotizing soft tissue infection in 161 Naja atra envenomed patients. PLoS neglected tropical diseases. 2022;16(2):e0010066.

4.	Chiang L-C, Tsai W-J, Liu P-Y, et al. Envenomation by Trimeresurus stejnegeri stejnegeri: clinical manifestations, treatment and associated factors for wound necrosis. Journal of Venomous Animals and Toxins including Tropical Diseases. 2020;26.

5.	Mao Y-C, Liu P-Y, Chiang L-C, et al. Clinical manifestations and treatments of Protobothrops mucrosquamatus bite and associated factors for wound necrosis and subsequent debridement and finger or toe amputation surgery. Clinical Toxicology. 2021;59(1):28-37.

6.	Soares Coriolano Coutinho JV, Fraga Guimarães T, Borges Valente B, et al. Epidemiology of secondary infection after snakebites in center-west Brazil. PLOS Neglected Tropical Diseases. 2023;17(3):e0011167.
