# Supplementary material for: Nationwide and long-term epidemiological research of snakebite envenomation in Taiwan during 2002–2014 based on the use of snake antivenoms: A study utilizing National Health Insurance Database
Source: PLoS Negl Trop Dis. 2023 Jun 8;17(6):e0011376. doi: 10.1371/journal.pntd.0011376 (PMC10249856; doi:10.1371/journal.pntd.0011376)
Supplement: Attachment — Submitted filename: 20230512 point to point response.docx [file pntd.0011376.s004.docx]

**Point to point response**

Dear Dr. Mao,
Thank you very much for submitting your manuscript "Nationwide and long-term epidemiological research of snakebite envenomation in Taiwan during 2002–2014 based on the use of snake antivenoms: a study utilizing National Health Insurance Database" for consideration at PLOS Neglected Tropical Diseases. As with all papers reviewed by the journal, your manuscript was reviewed by members of the editorial board and by several independent reviewers. The reviewers appreciated the attention to an important topic. Based on the reviews, we are likely to accept this manuscript for publication, providing that you modify the manuscript according to the review recommendations.
The revised manuscript satisfactorily responds to all the questions, queries and corrections suggested by the reviewers. Some parts of their rebuttal will help improve the manuscript. For example, segments in the rebuttal letter such as "We agree with you that diagnosing wound infections in SBEs can be difficult and may be overestimated if deep tissue or biopsy cultures are not performed for the wound. However, we observed that 76.8% to 80.9% of N. atra bite patients developed wound infections in studies conducted by Mao et al. Out of the 59 and 87 patients, 50 and 73, respectively, had positive wound bacterial cultures [1,2]. In addition, 22 out of 27 and 30 out of 33 had positive deep tissue or biopsy cultures, respectively [2,3]. Furthermore, necrotizing soft tissue infections, including necrotizing fasciitis, were observed in 42.1% to 44.7% of N. atra bites [2,3]. On the other hand, wound infections are less common in T. s. stejnegeri and P. mucrosquamatus (crotaline)
bites, with rates of 11.4% and 27.4%, respectively [4,5]. In this study, which utilized the National Health Insurance database, we used criteria similar to those in a recent report to identify SBE wound infections [6]. Our findings indicate that 20.3% of patients who received FH antivenom (i.e., antivenom for T. s. stejnegeri and P. mucrosquamatus) developed wound infections, which is consistent with previous studies conducted by Mao et al. [4,5], and lower than the study conducted by João Victor Soares Coriolano Coutinho et al. [6]. Although the incidence of wound infection might still have been overestimated in this study, such overestimation is much smaller" along with the references.

You could use such answers to reviewers' comments to improve the revised manuscript.
Please prepare and submit your revised manuscript within 30 days. If you anticipate any delay, please let us know the expected resubmission date by replying to this email. 
When you are ready to resubmit, please upload the following:
[1] A letter containing a detailed list of your responses to all review comments, and a description of the changes you have made in the manuscript.

Response: Thank you for the comments. We have added these sentences into the new 8^th^ paragraph in Discussion section: “Diagnosing wound infections in SBEs can be difficult and may be overestimated if deep tissue or biopsy cultures are not performed for the wound. However, we observed that 76.8% to 80.9% of N. atra bite patients developed wound infections in studies conducted by Mao et al. Out of the 59 and 87 patients, 50 and 73, respectively, had positive wound bacterial cultures [1,2]. In addition, 22 out of 27 and 30 out of 33 had positive deep tissue or biopsy cultures, respectively [2,3]. Furthermore, necrotizing soft tissue infections, including necrotizing fasciitis, were observed in 42.1% to 44.7% of N. atra bites [2,3]. On the other hand, wound infections are less common in T. s. stejnegeri and P. mucrosquamatus (crotaline) bites, with rates of 11.4% and 27.4%, respectively [4,5]. In this study, which utilized the National Health Insurance database, we used criteria similar to those in a recent report to identify SBE wound infections [6]. Our findings indicate that 20.3% of patients who received FH antivenom (i.e., antivenom for T. s. stejnegeri and P. mucrosquamatus) developed wound infections, which is consistent with previous studies conducted by Mao et al. [4,5], and lower than the study conducted by João Victor Soares Coriolano Coutinho et al. [6]. Although the incidence of wound infection might still have been overestimated in this study, we believed the overestimate was likely to be of limited magnitude.”

Please note while forming your response, if your article is accepted, you may have the opportunity to make the peer review history publicly available. The record will include editor decision letters (with reviews) and your responses to reviewer comments. If eligible, we will contact you to opt in or out
Response: Thank you for the suggestions. We agree to make the peer review history publicly available.

[2] Two versions of the revised manuscript: one with either highlights or tracked changes denoting where the text has been changed; the other a clean version (uploaded as the manuscript file).
Response: These are provided.

Important additional instructions are given below your reviewer comments.

Response: We have uploaded the data file for the figures.

**References**

1.	Mao YC, Liu PY, Hung DZ, et al. Bacteriology of Naja atra Snakebite Wound and Its Implications for Antibiotic Therapy. Am J Trop Med Hyg. 2016 May 4;94(5):1129-35.

2.	Mao Y-C, Liu P-Y, Chiang L-C, et al. Naja atra snakebite in Taiwan. Clinical Toxicology. 2018;56(4):273-280.

3.	Lai C-S, Liu P-Y, Lee C-H, et al. The development of surgical risk score and evaluation of necrotizing soft tissue infection in 161 Naja atra envenomed patients. PLoS neglected tropical diseases. 2022;16(2):e0010066.

4.	Chiang L-C, Tsai W-J, Liu P-Y, et al. Envenomation by Trimeresurus stejnegeri stejnegeri: clinical manifestations, treatment and associated factors for wound necrosis. Journal of Venomous Animals and Toxins including Tropical Diseases. 2020;26.

5.	Mao Y-C, Liu P-Y, Chiang L-C, et al. Clinical manifestations and treatments of Protobothrops mucrosquamatus bite and associated factors for wound necrosis and subsequent debridement and finger or toe amputation surgery. Clinical Toxicology. 2021;59(1):28-37.

6.	Soares Coriolano Coutinho JV, Fraga Guimarães T, Borges Valente B, et al. Epidemiology of secondary infection after snakebites in center-west Brazil. PLOS Neglected Tropical Diseases. 2023;17(3):e0011167.
